# Integrating Augmented Reality in Spine Surgery: Redefining Precision with New Technologies

**DOI:** 10.3390/brainsci14070645

**Published:** 2024-06-27

**Authors:** Manuel De Jesus Encarnacion Ramirez, Gennady Chmutin, Renat Nurmukhametov, Gervith Reyes Soto, Siddarth Kannan, Gennadi Piavchenko, Vladmir Nikolenko, Ibrahim E. Efe, Alberto Ramírez Romero, Jeff Ntalaja Mukengeshay, Keith Simfukwe, Tshiunza Mpoyi Cherubin, Federico Nicolosi, Salman Sharif, Juan Carlos Roa, Nicola Montemurro

**Affiliations:** 1 Department of Neurosurgery, Russian People’s Friendship University, 117198 Moscow, Russia; 2 Department of Head and Neck, Unidad de Neurociencias, Instituto Nacional de Cancerología, Mexico City 14080, Mexico; 3 School of Medicine, University of Central Lancashire, Preston PR0 2AA, UK; 4 Department of Human Anatomy and Histology, Sechenov University, 119911 Moscow, Russia; 5 Department of Neurosurgery, I.M. Sechenov First Moscow State Medical University (Sechenov University), 119991 Moscow, Russia; 6 Department of Neurosurgery, Charité—Universitätsmedizin Berlin, Corporate Member of Freie Universität Berlin, Humboldt-Universität zu Berlin, and Berlin Institute of Health, 10178 Berlin, Germany; 7 Department of Neurosurgery, Hospital Angeles Universidad, Mexico City 03330, Mexico; bto0@hotmail.com; 8 Department Neurosurgery, Clinique Ngaliema, Kinshasa 3089, Democratic Republic of the Congo; 9 Department of Medicine and Surgery, Neurosurgery, University of Milano-Bicocca, 20126 Milan, Italy; 10 Department of Neurosurgery, Liaquat National Hospital and Medical College, Karachi 05444, Pakistan; 11 Department of Pathology, School of Medicine, Pontificia Universidad Católica de Chile, Santiago 8330024, Chile; 12 Department of Neurosurgery, Azienda Ospedaliero Universitaria Pisana (AOUP), 56100 Pisa, Italy

**Keywords:** cervical spine, laboratory, 3D model training, residents, neurosurgery

## Abstract

Introduction: The integration of augmented reality (AR) in spine surgery marks a significant advancement, enhancing surgical precision and patient outcomes. AR provides immersive, three-dimensional visualizations of anatomical structures, facilitating meticulous planning and execution of spine surgeries. This technology not only improves spatial understanding and real-time navigation during procedures but also aims to reduce surgical invasiveness and operative times. Despite its potential, challenges such as model accuracy, user interface design, and the learning curve for new technology must be addressed. AR’s application extends beyond the operating room, offering valuable tools for medical education and improving patient communication and satisfaction. Material and methods: A literature review was conducted by searching PubMed and Scopus databases using keywords related to augmented reality in spine surgery, covering publications from January 2020 to January 2024. Results: In total, 319 articles were identified through the initial search of the databases. After screening titles and abstracts, 11 articles in total were included in the qualitative synthesis. Conclusion: Augmented reality (AR) is becoming a transformative force in spine surgery, enhancing precision, education, and outcomes despite hurdles like technical limitations and integration challenges. AR’s immersive visualizations and educational innovations, coupled with its potential synergy with AI and machine learning, indicate a bright future for surgical care. Despite the existing obstacles, AR’s impact on improving surgical accuracy and safety marks a significant leap forward in patient treatment and care.

## 1. Introduction

The evolution of medical technology has persistently sought to amalgamate accuracy with minimally invasive interventions, especially in fields requiring precision such as spine surgery. The spine, being a complex structure, commands absolute meticulousness and precision during surgical interventions due to its proximity to critical neural elements [1]. The integration of augmented reality (AR) in spine surgery has emerged as a groundbreaking advancement, paving the way for enhanced surgical precision and improved patient outcomes.

Augmented reality (AR), a technology that overlays computer-generated images on the user’s view of the real world, provides an immersive experience. This technology has demonstrated substantial promise in refining spine surgery procedures [2,3]. It enables surgeons to visualize anatomical structures and pathological entities in three dimensions, thereby offering a nuanced understanding and facilitating precise surgical planning and execution [3].

The incorporation of AR in spine surgery represents a paradigm shift in surgical methodologies, allowing for superior spatial understanding and real-time navigation during surgical procedures [4]. It has demonstrated potential in minimizing surgical invasiveness and reducing operative times, thereby contributing to improved postoperative recovery and reduced complication rates [5]. However, despite its promising applications, the integration of AR in spine surgery is accompanied by challenges and limitations. Issues related to the accuracy of AR models, user interface design, and the learning curve associated with mastering these advanced technologies underscore the need for comprehensive research and critical evaluation of AR applications in spine surgery [6,7]. In this era marked by rapid technological advancements, the exploration of augmented reality’s role in spine surgery is pivotal. It opens new vistas for surgical innovation, elevating the standards of surgical care, and pushing the boundaries of what is achievable in spine surgery [8,9]. The multidimensional visualization provided by AR aids in more precise differentiation between tissues, nerves, and bones, making intricate spinal surgeries more manageable and less prone to errors [10]. By allowing surgeons to interact with virtual representations of the spine, augmented reality fosters an environment where surgical strategies can be optimized, and potential complications can be anticipated and mitigated [11].

This transformative technology extends beyond operating rooms; it influences medical education and training, presenting aspiring surgeons with opportunities to gain experience and practice intricate spinal procedures in a simulated, risk-free environment [12]. The incorporation of AR in educational settings is proving instrumental in reducing the learning curve associated with complex spinal surgeries and facilitating the acquisition of essential surgical skills [13].

Moreover, AR’s scope in spine surgery contributes to improved patient communication and satisfaction. By utilizing AR, medical practitioners can better illustrate spinal conditions and proposed surgical interventions to their patients, enhancing patient understanding and involvement in their own healthcare journeys [14]. This improved patient-practitioner communication is integral in building trust and ensuring that patients are well informed and comfortable with the proposed surgical interventions [15].

The confluence of AR with other emerging technologies like artificial intelligence and machine learning opens possibilities for predictive analytics and personalized medicine in spine surgery [16]. This synergy could potentially lead to the development of sophisticated AR tools capable of providing real-time analytics and personalized data during surgeries, enabling surgeons to make more informed decisions and optimize surgical outcomes [17].

This literature review aims to explore the transformative role of augmented reality in spine surgery, delineating its current applications, significant benefits, and critical challenges. By examining recent studies and clinical trials, we seek to provide a comprehensive understanding of the current state of AR integration in spine surgery and its potential to revolutionize surgical precision in the coming years.

## 2. Materials and Methods

### 2.1. Search Strategy

To conduct a comprehensive literature review, a systematic search of the following electronic databases was performed: PubMed and Scopus. Keywords and MeSH (medical subject heading) terms used in the search included the following: “Augmented Reality” AND “Spine Surgery” OR “Spinal Procedures” AND “Surgical Navigation” OR “Medical Training” OR “Education” AND “Surgical Outcomes”.

The search strategy was designed to capture articles published in English from January 2020 to January 2024, ensuring the inclusion of the most recent and relevant studies. The search strategy aimed to identify a wide range of studies, including randomized controlled trials, observational studies, case studies, and cadaveric studies that investigated the applications, benefits, and challenges of augmented reality in spine surgery.

### 2.2. Study Selection

Initially, titles and abstracts were screened to identify articles that met the inclusion criteria. Inclusion criteria encompassed randomized controlled trials, observational studies, case studies and cadaveric studies that investigated the applications, benefits, and challenges of augmented reality in spine surgery. Exclusion criteria were studies not related to spine surgery, not utilizing augmented reality, containing less than 5 participants, with uncompleted data and that were non-English.

### 2.3. Data Extraction

From the selected studies, the following information was extracted: authors and year of publication, study design and sample size, AR technology utilized, applications of AR in spine surgery, methodology, sample size, primary outcomes, measures and limitations.

### 2.4. Analysis

A qualitative synthesis was performed to analyze the data extracted from the included studies. The analysis focused on assessing the impact of augmented reality on the precision, efficiency, and outcomes of spine surgery, its contribution to preoperative planning and intraoperative navigation, and its role in surgical education and training. Meta-analysis was not conducted due to the anticipated heterogeneity in study designs, AR technologies utilized, and reported outcomes.

### 2.5. Quality Assessment

The quality of the included studies was assessed using appropriate quality assessment tools, considering the study design. Randomized controlled trials were assessed using the Cochrane risk of bias tool, observational studies were assessed using the Newcastle–Ottawa scale, and case studies were evaluated based on their clarity, completeness, and methodological rigor.

### 2.6. Risk of Bias thorough Assessment of Risk of Bias Was Conducted in Included Studies, Considering Several Potential Sources of Bias

(1) Selection Bias: Selection bias was a concern due to the non-randomized selection of participants in case series and observational studies. Some studies had small sample sizes, which could limit the generalizability of the findings. The varying inclusion criteria across studies further contributed to the risk of selection bias.
(2) Performance Bias: Performance bias may have been introduced by the variability in the experience levels of surgeons and the learning curve associated with augmented reality technologies. The novelty of these technologies might have influenced performance outcomes, with some studies potentially favoring those more familiar with the technology.
(3) Detection Bias: Detection bias was considered due to the subjective nature of certain outcome assessments, such as user satisfaction and cognitive load. The use of non-standardized assessment tools across different studies may have further contributed to detection bias, impacting the reliability of the findings.
(4) Attrition Bias: Attrition bias was a potential issue as some studies did not report on the long-term follow-up of participants or provided incomplete data. The lack of comprehensive reporting on participant outcomes raises concerns about the robustness of the findings.
(5) Reporting Bias: Reporting bias was addressed by considering the possibility of selective publication of studies with positive outcomes. Negative or non-significant results might be underrepresented, skewing the overall assessment of augmented reality’s effectiveness in spine surgery.

Efforts were made to mitigate these biases through rigorous selection criteria, comprehensive quality assessment, and systematic data extraction and analysis. However, the inherent limitations in the available literature should be acknowledged, and the results of this review should be interpreted with these considerations in mind.

### 2.7. Ethical Consideration

As this study is a literature review, ethical approval and patient consent were not applicable. However, studies included in the review were appraised for ethical considerations, and the synthesis strictly adhered to the principles of research integrity and ethical reporting.

## 3. Results

### Study Selection

**Figure 1:**
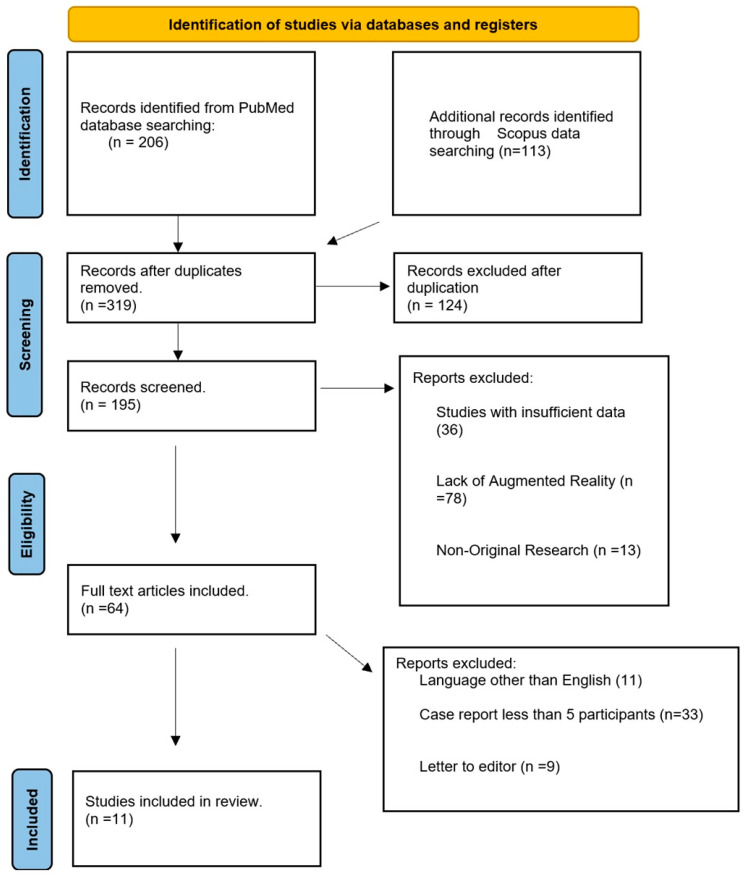
Illustrates the selection process of the studies included in the review.

Using the predefined search strategy, 319 articles in total were identified through the initial search of the databases. After removing duplicates, 195 articles remained. After screening titles and abstracts, articles that did not meet the inclusion criteria were excluded, and 64 full-text articles were assessed for eligibility. Following the full-text review, 11 articles were included in the qualitative synthesis. Figure 1 illustrates the selection process of the studies included in the review, while Table 1 provides a comparative overview of the articles analyzed.

## 4. Discussion

### 4.1. Applications of AR in Surgery

**Figure 2:**
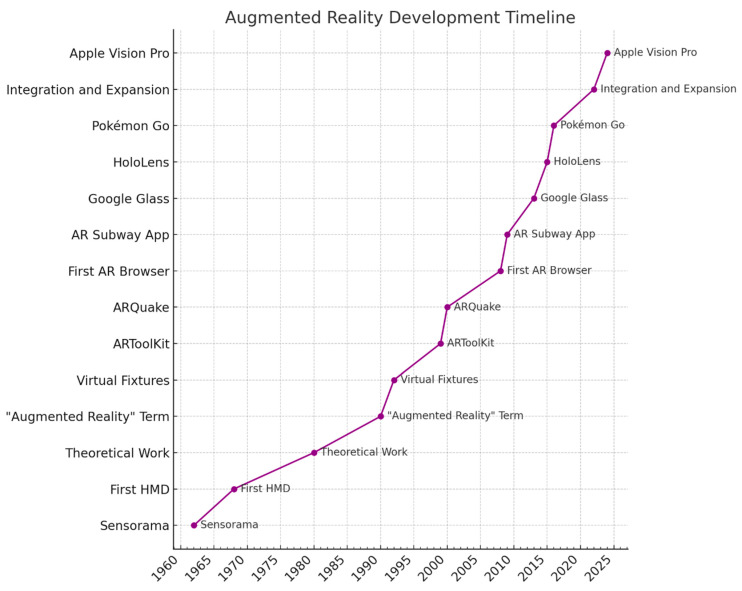
Technological evolution and impact.

The pioneering integration of AR within the medical field occurred during cranial neurosurgical operations in the 1980s [29]. This era witnessed the inception of the incorporation of image projecting systems into surgical microscopes, leading to the creation of microscope-based setups equipped with head-mounted displays (HMDs) and navigational technologies in the mid-1990s (Figure 2) [30]. By 1997, the initiative by Peuchot and colleagues introduced a system titled “Vertebral Vision with Virtual Reality”. This innovation enabled the overlay of three-dimensional, fluoroscopy-generated, transparent imagery of the spine onto the actual surgical view [31,32]. Progressively, AR technology evolved, allowing the seamless integration of intraoperative imagery or models with the live surgical environment [31]. Initially, this method marked a revolutionary step by facilitating the observation of spinal movements directly, thereby potentially reducing the need to consult external monitors and minimizing exposure to harmful ionizing radiation [32]. Research, including that by Molina et al. [32,33], highlighted the notably higher levels of ionizing radiation exposure during spinal surgery fluoroscopy compared with those in other medical subfields, underscoring the significance of AR in reducing radiation doses.

With the advent of AR technology becoming mainstream and the launch of HoloLens in 2016, an updated version, HoloLens 2, emerged, offering surgeons an enhanced capability to incorporate AR directly in the surgical field [34,35]. This device, worn over the head, provides transparent, floating images within the surgeon’s field of view, seamlessly integrating the patient’s anatomical images with the real-world surgery scene. Surgeons gained the flexibility to navigate around the patient, observing holographic images of internal body structures from various angles [33]. The system also supports voice and gesture commands for manipulating images or data, including the projection of vital signs into the surgeon’s visual field [36].

**Figure 3:**
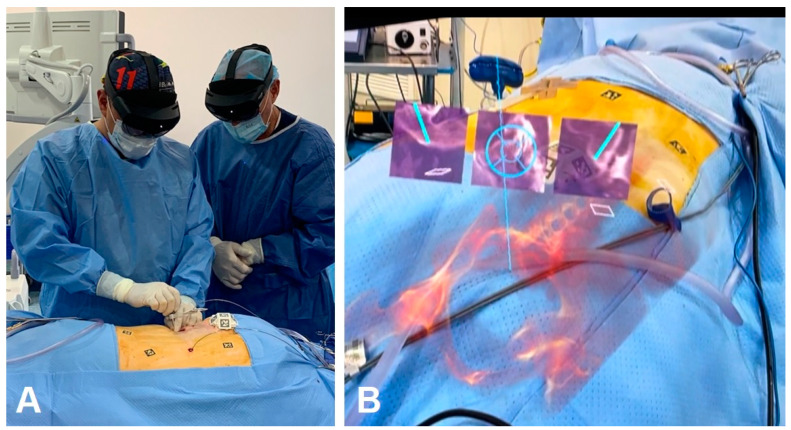
(**A**) Surgeon seeing a 3D image of the patient’s spine with two-dimensional CT images of the instruments’ path and trajectory while looking directly at the surgical field; (**B**) 3D visualization of the surgeon’s retina. Authors’ original files.

**Figure 4:**
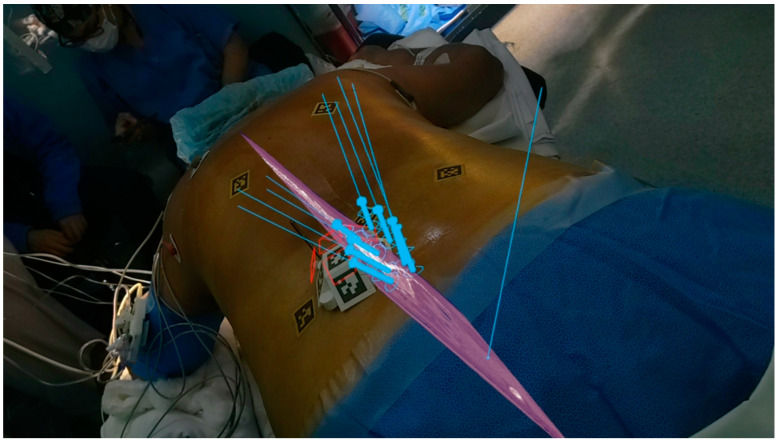
Surgeon looking through the Hololens 2 headset demonstrating virtual three-dimensional projection and two-dimensional cross-sectional navigation cuts.

The landmark AR-assisted spinal surgery on a living patient was conducted in 2020 using the XVision system (Figure 3 and Figure 4) by Au medics at John Hopkins University including Timothy Witham [37,38]. This historical operation, performed on 8 June 2020, involved inserting six screws in a spinal fusion procedure to alleviate chronic back pain by fusing three vertebrae [37]. A subsequent operation on June 10 removed a malignant spinal tumor [37]. Preceding these surgeries, in 2019, Frank Phillips at Rush University published a study on AR-assisted pedicle screw insertion using cadavers. Following this, Phillips executed the inaugural AR-guided minimally invasive spine surgery (MISS) using the same XVision system within the next year [38]. This procedure entailed performing a lumbar fusion to treat a patient with spinal instability, during which the AR headset projected a 3D navigational map directly onto the surgeon’s retina. This enabled the visualization of the patient’s spine in three dimensions with the skin unopened, alongside two-dimensional (2D) CT scans displaying the surgical instruments’ intended paths; see Figure 4 [35].

**Figure 5:**
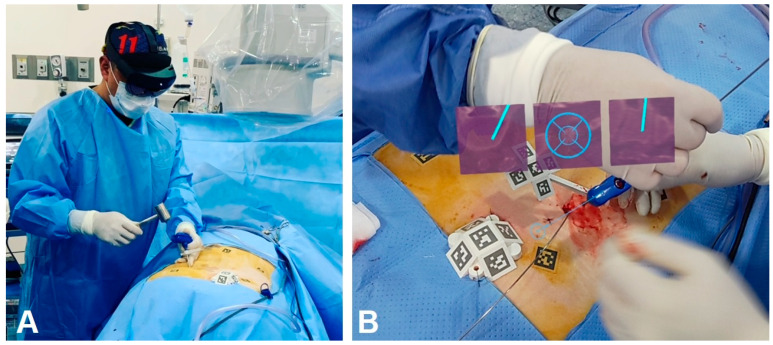
MISS navigation procedure utilizing AR Hololens 2 technology. (**A**) AprilTags are adhered to the skin and also placed on platforms stabilized by bone pins. The Jamshidi needle is aligned with the virtual needle/pathway and has been inserted percutaneously. (**B**) Lateral and axial view of a MISS procedure in progress under Hololens navigation. Note the Jamshidi needle that has successfully penetrated the underlying pedicle. The center of the needle has been extracted, and a K-wire has been inserted for guidance of a cannulated screw. An optical fiducial (AprilTag) appears below the vertebrae. MISS, minimally invasive spine surgery.

The advent of AR in spine surgery ushers in a new era of medical innovation, transforming not only how surgical procedures are performed but also how they are conceptualized from the planning stages through to post-operative care. AR technology, through its integration with devices like Microsoft HoloLens, (Figure 5) and AR-Virtual Needle (VN), is at the forefront of enhancing surgical precision, optimizing the learning process for medical professionals, and significantly improving patient outcomes.

### 4.2. Technological Evolution and Impact

Microsoft HoloLens: By providing immersive, three-dimensional visualizations of the surgical field, HoloLens enhances spatial awareness and operational precision. This technology overlays critical digital information over the physical world, enabling surgeons to navigate complex anatomical structures with enhanced visibility and accuracy, thus reducing the risk of surgical errors and improving patient safety [30].

AR Goggles and Glasses: These devices extend the application of AR technology to the entire surgical team, fostering an environment of collaborative expertise and remote assistance. This collaboration is crucial in complex surgical scenarios where multi-disciplinary input is vital for successful outcomes, thereby streamlining surgical processes and enhancing the overall quality of patient care [25].

The integration of AR with AI and machine learning algorithms marks a significant leap forward, offering predictive analytics and personalized surgical planning [39]. This confluence of technologies can analyze extensive data sets to deliver tailored insights and recommendations, enabling a more targeted and effective surgical approach. Such capabilities are crucial in navigating the complexities of spinal surgeries, where precision is paramount [40].

Preoperative Planning: The ability to generate detailed, patient-specific 3D models allows for unparalleled surgical planning. This meticulous preparation is instrumental in foreseeing potential obstacles and crafting a strategic approach that is customized to each patient’s unique anatomy, thereby enhancing the likelihood of a successful surgical outcome [38].

Intraoperative Navigation: During the procedure, AR offers real-time navigational assistance, superimposing essential data directly into the surgeon’s field of view. This innovation is pivotal in performing accurate surgical interventions, as evidenced by the improved placement of pedicle screws and the minimized risk of complications [26].

Educational Enhancement: AR transforms surgical training by simulating complex procedures in a risk-free environment. This application significantly reduces the learning curve for intricate spinal surgeries and fosters a deeper understanding of spinal anatomy and surgical techniques, thereby advancing the proficiency and confidence of emerging surgeons [27].

### 4.3. Research and Development

The exploration of AR in spine surgery is supported by a diverse range of research methodologies, encompassing literature and systematic reviews, clinical studies, and experimental research. This comprehensive approach enables a thorough understanding of AR’s effectiveness, safety, and areas for improvement, contributing to the ongoing refinement and adoption of AR technologies in surgical practices [1,41,42] (Table 2).

### 4.4. Learning Curve

AR technology has a long history, yet recently, it has captured renewed attention, especially in minimally invasive spinal surgery (MISS) [49]. The application of these innovative AR tools is still in its infancy in operating rooms globally, but there is a strong interest in leveraging their potential. When integrating new AR systems into surgical procedures, challenges can arise either before or during the operation. An instance of such challenge was highlighted by Urakov et al. [50], reporting an unexpected shutdown of their AR software 1.0, which resulted in extended surgery times. Additionally, the intricacies of AR spinal navigation (ARSN) and the latest augmented display head-mounted devices (AD-HMDs) might discourage experienced surgeons from adopting these technologies in their routine practices [51]. The introduction of AR innovations necessitates a significant learning period for the medical team, including surgeons, nurses, and technical staff. Nonetheless, data indicate that surgical techniques and patient outcomes improve for those who consistently utilize AR technologies. Gasco et al. [52] demonstrated that using AR as an educational resource could reduce errors by approximately 50% compared with traditional visual and verbal training methods. Similarly, employing AR for MISS education can potentially reduce the training period, enabling healthcare institutions that invest in AR to efficiently train more surgeons in MISS methodologies and reap financial benefits [53,54,55,56].

### 4.5. Looking Ahead

As AR technology continues to evolve, its potential applications within spine surgery and beyond are vast and varied. Future advancements may include more sophisticated AI integration for even more precise surgical planning and real-time decision support, as well as the development of haptic feedback systems for an enhanced sense of touch in virtual environments. Such innovations could further reduce operative times, improve surgical outcomes, and enhance the educational experience for surgical trainees.

The integration of 3D printing with augmented reality (AR) in spine surgery represents a significant evolution in how surgeons plan and execute procedures. Utilizing patient-specific 3D printed models offers tangible, accurate replicas of patient anatomy, enhancing both preoperative planning and intraoperative guidance [57]. These models provide a tactile, comprehensive understanding of complex anatomical structures, which is crucial for surgeries requiring high precision, such as those involving the spine [58,59,60].

When combined with AR, 3D-printed models can be superimposed with dynamic digital information, such as nerve paths and vascular structures. This overlay enhances surgeons’ comprehension and planning accuracy, ensuring a thorough consideration of the patient’s unique anatomy [61]. During surgery, AR projects this enhanced data directly onto the surgeon’s field of view, facilitating a real-time comparison between the model and the actual surgical site. This capability is especially vital for tasks requiring high precision, like the placement of screws or custom implants [62,63]. Moreover, the use of AR and 3D models extends beyond the operating room. After surgery, these models prove invaluable for validating surgical outcomes and explaining the procedures to patients, which can significantly enhance patient understanding and satisfaction. The combination of visual and tactile elements helps demystify the surgical process, reducing post-operative anxiety and potentially leading to better recovery outcomes [64,65].

In recent innovations, the employment of AR with 3D-printed models in spine surgeries has been documented from preoperative phases, where meticulous planning is enhanced, through to the surgical procedures where AR guides implementation, and postoperative assessments comparing actual outcomes with initial plans [66,67]. Clinical trials and case studies have shown promising results in implant accuracy, reductions in surgery times, and improvements in patient recovery times and satisfaction [68].

Looking ahead, the potential for technological advancements to further enhance the synergy between 3D printing and AR is vast [69]. Automated adjustments to models based on real-time feedback, integration with machine learning to optimize surgical strategies, and improvements in training programs for new surgeons are all areas ripe for development [68,70]. These advancements could streamline surgical procedures and increase success rates, making a profound impact on both surgical outcomes and the training of future surgeons [71,72]. However, a notable hurdle in the widespread adoption of such technologies is their cost. The expense associated with implementing and maintaining state-of-the-art AR systems can be prohibitive, limiting access primarily to well-funded healthcare institutions. This financial barrier may slow the integration of AR into widespread clinical use, despite its potential to revolutionize surgical practices and educational methods [73,74,75,76,77].

As AR technology continues to evolve, its potential applications within spine surgery and beyond are vast and varied. Several specific advancements and research areas hold promise for the future.

### 4.6. Integration with AI and Machine Learning

Real-Time Decision Support: The integration of AI and machine learning can enhance AR systems by providing real-time analytics and decision support during surgery. AI algorithms can analyze vast amounts of surgical data to offer personalized recommendations and predictive insights, improving surgical outcomes and reducing complications [78,79]. The use of telemedicine, with its limitations, could help in this [80].

Predictive Analytics: AI-driven predictive analytics can help identify potential issues before they arise, enabling surgeons to plan and execute procedures with greater precision and confidence. This capability is particularly valuable in complex spinal surgeries, where anticipating challenges can significantly impact success rates [81,82].

### 4.7. Haptic Feedback Systems

Enhanced Sensory Input: The development of haptic feedback systems can provide surgeons with tactile sensations that mimic the feeling of actual tissues and structures during surgery. This advancement would enhance the realism of AR simulations and improve the surgeon’s ability to perform delicate maneuvers with precision [83,84]. Training and Skill Development: Haptic feedback can also play a crucial role in surgical training, allowing trainees to experience the tactile aspects of surgery in a risk-free environment. This can accelerate the learning curve and improve the overall proficiency of emerging surgeons [58,76,85].

### 4.8. Critical Analysis of Overall Findings

The reviewed studies consistently highlight the significant potential of AR in enhancing the precision and efficiency of spine surgery. Common findings across the studies include the following:

(1) Improved Surgical Outcomes: Several studies, such as those by Xin et al. [19] and Edström et al. [28], reported improved accuracy in procedures like pedicle screw placement and reduced attention shift for surgeons. These improvements can lead to better surgical outcomes and reduced complications.
(2) Enhanced Surgical Education: The use of AR for surgical training and education was a prominent theme. Studies like those of Babichenko et al. [18] and Schonfeld et al. [25] highlighted AR’s role in reducing cognitive load and enhancing the learning experience for surgeons, which can lead to improved skill acquisition and performance.
(3) Efficiency Gains: Several studies reported efficiency gains, such as reduced operative times and improved workflow. For example, DeSalvatore et al. [26] found that using AR technology decreased operative time and bleeding while increasing surgeon satisfaction.
(4) Patient Outcomes and Satisfaction: The potential for AR to improve patient communication and satisfaction was noted, as it allows surgeons to better explain procedures and expected outcomes. This was particularly highlighted in studies like those by Rush et al. [27], which demonstrated the benefits of AR in preoperative planning and patient education.

Despite these positive findings, discrepancies exist, particularly regarding the extent of AR’s impact on surgical performance and patient outcomes. Some studies reported significant improvements, while others, like that of Pojskić et al. [21], indicated that AR’s benefits might not always translate into clinically significant differences.

### 4.9. Specific Challenges

While AR technology offers many benefits, several challenges and limitations must be addressed:

(1) Technological Barriers

Image Latency and Quality: Ensuring real-time, high-quality imaging without latency is crucial for effective AR-assisted surgery. Current systems sometimes struggle with image lag and resolution issues, which can impede surgical precision [86].

User Interface Design: The complexity of AR interfaces can be a barrier, necessitating intuitive designs that minimize cognitive load and maximize ease of use for surgeons [87].

(2) Cost Issues

High initial Investment: The significant upfront costs for AR systems, such as those of Augmedics XVision, the newly released Apple Vision Pro in 2024 and Microsoft HoloLens Table 2, can be prohibitive for many healthcare institutions. Ongoing costs for maintenance, updates, and training further add to the financial burden [38,43,44,45,46,47,48].

Software Integration Costs: Beyond hardware costs, the high price of the software necessary to integrate AR devices with patient data for surgical use can be a significant financial barrier. This includes expenses for acquiring, customizing, and maintaining software systems that ensure the seamless and secure integration of patient data with AR platforms [88].

Cost–Benefit Analysis: A comprehensive cost–benefit analysis is essential to justifying the investment in AR technologies, especially in resource-limited settings. Demonstrating potential savings from reduced operative times, improved surgical outcomes, and enhanced training is crucial [89].

### 4.10. Limitations of This Study

Limited scope of literature: The review’s scope is confined to articles published in English between 1 January 2020 and January 2024, potentially omitting valuable research and developments in AR for spine surgery published outside this timeframe or in languages other than English.

Heterogeneity in study designs: The included studies exhibit a broad range of methodologies, from case studies and observational studies to clinical trials. This diversity, while enriching, makes it challenging to conduct a uniform analysis or a meta-analysis, limiting the ability to generalize findings across different settings and populations.

Quality and bias in selected studies: The quality assessment of included studies relied on tools appropriate to each study design. However, the inherent bias within individual studies, such as selection bias or publication bias, may influence the overall conclusions drawn from this literature review.

Technological variability: The AR technologies discussed in the study, including Microsoft HoloLens, AR goggles, and the AR-Virtual Needle, vary significantly in their design, application, and maturity. This variability can lead to differences in user experience, efficacy, and outcomes, which are not uniformly addressed in the review.

Implementation and integration challenges: While the review acknowledges the potential of AR, it may underrepresent the practical challenges of integrating AR technologies into existing surgical workflows and systems. Issues such as compatibility with current medical devices, operating room logistics, and staff training requirements are critical for successful implementation but are not extensively discussed.

User experience and learning curve: The study mentions the learning curve associated with mastering AR technologies but does not delve into the specifics of user experience, including the ergonomics of AR devices, user interface design, and the cognitive load on surgeons. These factors are crucial for the adoption and effective use of AR in clinical settings.

Cost-effectiveness analysis: The review lacks a detailed cost–benefit analysis of AR technology integration into spine surgery. The high costs associated with AR equipment, software development, and maintenance could be a significant barrier to its widespread adoption, especially in resource-limited settings.

Ethical and legal considerations: While the study briefly touches upon ethical considerations, there is a lack of comprehensive analysis on ethical and legal implications, including patient consent for the use of AR, data privacy issues, and liability in case of technology failure or surgical complications.

Patient-centered outcomes: The primary focus of the study is on surgical precision and efficiency. There is limited discussion on patient-centered outcomes, such as postoperative pain, long-term recovery, and patient satisfaction, which are critical for evaluating the overall benefit of AR technologies in spine surgery. It is crucial to acknowledge that while Apple Vision Pro was employed during spine surgery, comprehensive data collection on its performance and outcomes was not feasible. This single instance of its use in a clinical setting does not provide a sufficient evidence base to conclusively evaluate its effectiveness, potential benefits, or drawbacks within the context of spinal operations. Consequently, our analysis lacks detailed insights into how Apple Vision Pro might impact clinical outcomes, efficiency, or cost-effectiveness in surgical procedures when compared with other augmented reality technologies. Further research and more extensive clinical trials are necessary to fully ascertain the capabilities and limitations of Apple Vision Pro in medical applications.

## 5. Conclusions

The study on the integration of AR in spine surgery reveals a promising advancement in medical technology, with the potential to significantly improve surgical precision, training, and patient outcomes. Despite challenges such as technological limitations, integration complexities, and the need for extensive training, AR offers a novel way to enhance surgical accuracy and safety. It provides immersive visualizations that aid in complex procedures and introduces innovative methods for surgical education. Looking ahead, the fusion of AR with technologies like AI and machine learning promises to push the boundaries of surgical care further. However, the high costs associated with AR systems pose a substantial barrier, potentially impeding their widespread adoption despite their revolutionary potential in surgery and training methods.

## Figures and Tables

**Table 1:** Provides a comparative overview of the articles analyzed.

Author(s) (Year)	Objective	AR Technology	Applications of AR in Spine Surgery	Methodology	Primary Findings	Sample Size	Primary Outcomes and Measures	Limitations
Babichenko et al. (2023) [18]	Systematically collect and examine the role of AR in spine surgery. aims to highlight the evolution of AR technology in this context, evaluate the existing body of research, and outline potential future directions for integrating AR into spine surgery.	Microsoft HoloLens	It highlights the strengths and weaknesses of existing investigations. Additionally, it presents insights into the potential for AR to enhance spine surgical education and speculates on future applications.	Case series	No significant differences presented in cognitive load when trials with the HoloLens 1 in comparison with the trials without the HoloLens 1. Cognitive load was measured with the Surgical Task Load Index (SURG-TLX) questionnaire and surgical performance metrics.	22 Surgeons participated	The results from our literature review provide a centralized pool of in vivo metrics related to AR, virtual reality (VR), and mixed reality (MR) use in spine surgery.	Limited studies have been reported regarding the clinical results of AR, VR, and MR use in live surgery of the spine.
Xin et al., 2020 [19]	It shows the current evidence of the use of VR, AR, and MR simulators in minimally invasive spine surgery (MISS) and spinal endoscopic surgery, including study quality, level of evidence (LoE), and outcomes.	Phantom	Screw placement	RCT	To verify whether the pedicle screw placement (PSP) skills of young surgeons receiving immersive virtual reality surgical simulator (IVRSS) training could be improved effectively and whether the IVRSS-PSP training mode could produce real clinical value for clinical surgery.	24 participants	The current scope of VR, AR, and MR surgical simulators in MISS was described.	A lack of clearly defined outcomes, absent statistical analyses, limited validity breadth including demonstration of transfer validity
Buch et al. (2021) [20]	Innovative solutions can be developed to enable the use of this technology during surgery.	HoloLens	3D visualization,	Experiment	The pipeline uses intraoperatively acquired, low-resolution imaging to generate, deploy, and register holographic models onto patients on the operating table	16 participants	A custom pipeline is described for the generation of intraoperative 3D holographic models during spine surgery.	More testing is required to confirm clinically adequate registration accuracy across multiple patients
Pojskić et al. (2024) [21]	Single center experience in resection of intradural spinal tumors either with or without using intraoperative CT (iCT)-based registration and microscope-based augmented reality (AR). Microscope-based AR was recently described for improved orientation in the operative field in spine surgery, using superimposed images of segmented structures of interest in a two- (2D) or three-dimensional (3D) mode	based registration and microscope-based augmented reality (AR)	resection of intradural spinal tumors	Case series	Its key advantage over robotics and navigated spine surgery is that the surgeon never has to take the focus from the patient	112 patients	Operative time, extent of resection, clinical outcome and complication rate did not differ between the AR and non-AR group. However, use of AR improved orientation in the operative field by identification of important neurovascular structures. AR improved intraoperative orientation and increased surgeons comfort by enabling early identification of important anatomical structures, However, clinical and radiological outcomes did not differ, when AR was not used.	The cost of technology and justification of its use without differences in clinical and surgical outcomes and complication rates
Charles et al. (2022) [22]	To assess intra- and inter-observer reliability of pedicle screw placement and to compare the perception of baseline image quality (NoMAR) with optimized image quality (MAR).	Microsoft Holo Lens	Microscopic surgery, remote assistance	Case series	Assessment for accuracy of pedicle screw placement.	24 patients	Intraoperative screw positioning can be reliably assessed on cone beam CT for AR surgical navigation when using optimized image quality. MAR and NoMAR images demonstrated good intra-observer and excellent inter-observer and intra-class correlation coefficients.	Various AR-enabled technologies are emerging without specific criteria for judging them.
Carl et al. (2020) [23]	To investigate how microscope-based augmented reality (AR) support can be utilized in various types of spine surgery.	Microscope-based AR support	The application of AR technology in various facets of spine surgery. The included studies illustrate the potential benefits and feasibility of utilizing AR in spine surgical procedures, highlighting its impact on patient outcomes and surgeon performance.	Case series	Anterior, lateral, posterior median, and posterior paramedian approaches for degenerative spine surgery as well as intradural and extradural tumor resections.	42 patients (12 intra- and 8 extradural tumors, 7 other intradural lesions, 11 degenerative cases, 2 infections, and 2 deformities). AR was implemented using operating microscope head-up displays (HUDs)	AR smoothly supported various kinds of spine procedures and facilitated anatomical orientation in the surgical field.	It is difficult to exactly measure the additional benefit of the AR application in each individual procedure. Usability questionnaires might be a tool to document surgeon acceptance
Mozaffari et al. (2020) [24]	To investigate how microscope-based augmented reality (AR) support can be utilized in traumatic spine surgery and focal kyphosis.	Microscopic-AR, VisAR	Microscope-based AR can be applied successfully to various kinds of spinal procedures. AR improves anatomical orientation in the surgical field, supporting the surgeon, as well as offering a potential tool for education.	Clinical study	The entire process of intraoperative registration. Imaging was added only at about 5 min into the surgical procedure, and thereafter, AR was instantly available. We did not encounter any technical or surgical problems due to AR implementation.	10 patients	A microscope-based AR environment was successfully implemented for spinal surgery. The application of Ict for registration imaging ensures high navigational accuracy. AR supports the surgeon in understanding the 3D anatomy, thereby facilitating surgery.	Among the limitations of our study is that it is difficult to exactly measure the additional benefit of the AR application in each individual procedure. Usability questionnaires might be a tool to document surgeon acceptance.
Schonfeld et al. (2021) [25]	Analyzed several scenarios where we equipped OR personnel with augmented reality (AR) glasses, allowing a remote specialist to guide OR operations through voice and ad hoc visuals, superimposed to the field of view of the operator wearing them.	AR goggles, XVision	Remote assistance	Clinical study	Surgeons wearing the AR goggles reported positive feedback as for the ergonomics, wearability, and comfort during the procedure; being able to visualize a 3D reconstruction during surgery was perceived as a straightforward benefit, allowing surgeons to speed-up procedures, thus limiting post-operational complications.	21 participants	By allowing surgeons to overlay digital medical content on actual surroundings, augmented reality surgery can be exploited easily in multiple scenarios by adapting commercially available or custom-made apps to several use cases.	Physical limitations, limited exploration of dose ranges, and denoising algorithm artifacts focus solely on AR auto-segmentation, potential impact on clinical workflow, and limited generalizability to diverse clinical environments and AR systems.
DeSalvatore et al. (2020) [26]	To assess a novel 3D model created using Google Cardboard for surgical planning for adolescent idiopathic scoliosis patients.	AR goggles, Google Cardboard	Intraoperative Navigation	Case series	The main findings were superior workflow and non-inferior accuracy when comparing AR to free-hand (FH) or conventional surgical navigation techniques.	60 patients	Use of this VR-based technology led to decreased operative time and bleeding while increasing the surgeon’s satisfaction in a reproducible, cost-effective manner.	The current evidence base is limited and prospective studies on clinical outcomes and cost–benefit relationships are needed.
Rush et al. (2022) [27]	Augmented reality (AR) has the potential to dramatically improve the accuracy and reduce the time required for preoperative planning and performance of minimally invasive spine surgeries and procedures.	XVision	AR surgical navigation	Cadaveric study	This data set suggests that AR navigation, utilizing a VN, is an emerging, accurate, valuable additive method for surgical and procedural planning for percutaneous image-guided spinal procedures and has potential to be applied to a broad range of clinical and surgical applications.	5 cadavers, 120 screws	application of AR navigation on a series of common percutaneous image-guided spine procedures	Physical limitations, limited exploration of dose ranges, denoising algorithm artifacts, focus solely on AR auto-segmentation, potential impact on clinical workflow, and limited generalizability to diverse clinical environments and AR systems.
Edström et al. (2020) [28]	To present a workflow for an ARSN system installed in a hybrid operating room.	augmented-reality-based surgical navigation (ARSN) system installed in a hybrid operating room	Pedicle screw placement.	Case series	Microscope AR displays offer the advantage of reducing attention shift and line-of-sight interruptions inherent in traditional instruments.	20 cases	Navigated interventions were performed with a median total time of 379 min per procedure. The total procedure time was subdivided into surgical exposure (28%), cone beam computed tomography imaging and 3D segmentation (2%), software planning (6%), navigated surgery for screw placement (17%), and non-navigated instrumentation, wound closure (47%).	Limited clinical results, intraoperative CT scan required, registration markers unavailable for certain rigid locations.

**Table 2:** Current trending AR technologies used in medicine.

Technology	Characteristics	Outcomes in Clinical Settings	Pricing
Augmedics XVision	Retina-projecting heads-up display, incorporating 3D anatomical precision and tool details, surgical navigation, integrated illumination, high-speed visual processing, cordless	Studies on cadavers: 94.6–98.9% precision according to the Gertzbein scale, and 96.7–99.1% according to the Heary–Gertzbein scale. Patient studies: 98.0–100% accuracy [38,43,44]	USD 200,000 (2020)
Microsoft HoloLens	Cordless headset with holographic lenses, quad visible light and dual IR cameras, integrated microphones and speakers, Bluetooth connectivity, Wi-Fi	Cadaver studies: Precision on par with top-tier tracking systems. Phantom studies: Reduced total time for rod bending and insertion by 20% [4,45,46]	USD 3500–USD 5200 (2023)
Applevision pro	Oculus-based headset with holographic lenses, precision measurement, adjustable opacity, sonic feedback, user-friendly menus and buttons	First case in the world registered using vision pro in ventricular catheter placement, good results not complications, first spine surgery case [47,48]	USD 3500 (2024)
